# STRIDE: a randomized trial of a lifestyle intervention to promote weight loss among individuals taking antipsychotic medications

**DOI:** 10.1186/1471-244X-13-238

**Published:** 2013-09-28

**Authors:** Bobbi Jo H Yarborough, Michael C Leo, Scott Stumbo, Nancy A Perrin, Carla A Green

**Affiliations:** 1 Kaiser Permanente Center for Health Research, 3800 N. Interstate Avenue, Portland, OR 97227, USA

**Keywords:** Antipsychotic medications, Serious mental illness, Obesity, Lifestyle change, Weight loss, Physical activity, Diabetes risk, Blood pressure, Lipids

## Abstract

**Background:**

Individuals diagnosed with serious mental illnesses are at increased risk of obesity- and cardiovascular-related morbidity and early mortality. Lifestyle interventions aimed at weight loss, even those adapted to suit the needs of this particular subgroup, have rarely produced clinically meaningful reductions in weight.

**Methods/design:**

The STRIDE study is a multi-site, parallel, two-arm randomized controlled translational trial. Participants were recruited from community mental health clinics and an integrated not-for-profit health system. Participants were randomized either to usual care or to a 12-month intervention that consisted of: 1) weekly group participation for six months covering topics on nutrition, physical activity and lifestyle changes; 2) monthly group participation for an additional six month maintenance period; and 3) individual monthly contacts from intervention group facilitators during the second six month phase. All participants are assessed at baseline, 6, 12, and 24 months post-enrollment. Process and implementation evaluations are included and the study design includes a cost-utility analysis. Participants include 200 individuals with serious mental illness with an average age of 47.1 years, a mean body-mass index of 38.3 kg/m^2^ and taking an average of 3.2 psychiatric medications at baseline. Baseline physiological measures included mean blood pressure (SBP/DBP) measurements of 119.2 (SD = 14.7)/79.4 (SD = 10.1); 35% reported a hypertension diagnosis and 11% took antihypertensive medications. Average lipid levels (mg/dL) were: a) triglycerides 188.0 (SD = 138.6), ranged from 43 to 1145; b) LDL 101.4 (SD = 32.9) and ranged from 17 to 185; c) HDL 45.8 (SD = 12.7) and ranged from 22 to 89; and d) total cholesterol 181.6 (SD = 39.7) and ranged from 50 to 324. Average fasting glucose levels were 108.9 (SD = 32.5) and ranged from 24 to 289. Average fasting insulin levels were 13.0 (SD=11.9) and ranged from 2 to 99.

**Discussion:**

The STRIDE study is based on a modified version of the PREMIER comprehensive lifestyle intervention, DASH diet arm. STRIDE has successfully enrolled 200 individuals with serious mental illness in community-based settings. Baseline characteristics present a population at high risk for obesity-related negative health outcomes and demonstrate the need for evidence-based interventions to reduce these risks.

**Trial registration:**

Clinical Trials.gov NCT00790517

## Background

Individuals with serious mental illnesses are at greatly increased risk of a number of medical comorbidities including obesity [1], metabolic syndrome [2-4], diabetes mellitus [4-6], and subsequent early mortality [7-9], primarily from cardiovascular disease [2,10,11]. The causes of these elevated cardiometabolic risks can include factors such as poor access to medical care [12,13], poor nutrition [14], sedentary lifestyle [11], and smoking [11,15] but they can also be exacerbated by the antipsychotic agents prescribed to treat mental health conditions [16-23]. Because many individuals diagnosed with serious mental illnesses rely on antipsychotic medications as a primary component of their treatment, complementary treatments that reduce weight gain and potentially reduce associated cardiometabolic risks have been suggested by clinicians and researchers [1]. Such treatments appear feasible [24,25] and valued by potential recipients [25] but their effectiveness has been inconsistent and, when effective, weight losses have been modest [26]. Bartels and Desilets found that manualized lifestyle health promotion programs lasting more than 3 months—combining activities with education, emphasizing weight management through improved nutrition and physical exercise—promote success but nevertheless only result in clinically significant weight loss for a minority of individuals [26]. Community-based approaches which improve the magnitude of weight loss and physical fitness are needed as well as interventions that reduce cardiometabolic risks, e.g., blood pressure, insulin resistance, or lipid levels.

This protocol describes a randomized controlled trial assessing the effectiveness of translating a known efficacious and comprehensive lifestyle intervention, adapted for individuals with serious mental illnesses at high risk for obesity, diabetes, and cardiovascular disease, and receiving community-based treatment, including antipsychotic medications, for their mental health disorders.

## Methods/design

The STRIDE study is a parallel-arm, multi-site, randomized controlled trial, translating the PREMIER comprehensive lifestyle intervention [27-30] DASH diet arm [31] for individuals taking antipsychotic medications. The primary aims are to test the hypotheses that the intervention is more effective than usual care in: 1) reducing weight and body mass index (BMI); 2) reducing fasting insulin levels and increasing insulin sensitivity; and 3) reducing total cholesterol and LDL cholesterol at 6, 12, and 24 months. Secondary aims are to explore the effects of exercise motivation, dietary motivation, social support and weight loss expectations on primary outcomes, and to examine the moderating effects of ethnicity, gender, mental health diagnostic group, medication type, and metabolic syndrome. Process and implementation evaluations are designed to identify facilitators and barriers of behavior change among participants; a cost-utility analysis will provide economic information for program planners considering adoption.

### Settings

To increase clinical relevance and the likelihood of future adoption, implementation, and sustainability of the intervention, we limited our exclusion criteria and worked with community partners to deliver the intervention in routine care settings. Our partners include two community mental health centers—Cascadia Behavioral Healthcare (Cascadia) and LifeWorks Northwest (LifeWorks)—and Kaiser Permanente Northwest (KPNW), a not-for-profit integrated health system. Cascadia serves approximately 12,000 low-income clients by providing outpatient mental health care and addiction treatment services across three Oregon counties. LifeWorks serves the mental health and addiction treatment needs of approximately 16,000 clients annually in 22 clinics throughout the greater Portland, Oregon metropolitan area. The majority of these clients come from impoverished and underserved segments of the population. KPNW provides comprehensive medical, mental health and addiction treatment to an insured population of about 480,000 members in northwest Oregon and southwest Washington. All study procedures were reviewed, approved, and are monitored by the Kaiser Permanente Northwest Institutional Review Board (other participating sites ceded authority to KPNW). All participants were informed of their rights and responsibilities as research participants and provided written informed consent prior to enrollment.

### Participants

Our sample includes individuals age ≥18 who were taking antipsychotic agents at study enrollment and who were overweight or obese (defined as BMI ≥ 27). We asked prescribing clinicians to review records for potential participants to ensure that they did not have medical or psychiatric conditions that precluded participation in a lifestyle and weight loss program that included changes to dietary practices and promotion of moderate exercise. We excluded individuals with cognitive impairment sufficient to interfere with their ability to provide informed consent, complete study questionnaires, or participate in a group intervention. We excluded women who were pregnant, breastfeeding, or planning a pregnancy during the study because reduced calorie intake may be contraindicated. We also excluded people with a bariatric surgery history or a wasting illness (e.g., cancer) because they are at increased risk for weight loss as a result of their medical status. Finally, we excluded individuals currently enrolled in another weight reduction program as inclusion could confound the results of our intervention.

### Recruitment, screening, and randomization

Using administrative and clinical databases at two sites (KPNW, Cascadia) and mental health clinician referral at the third site (LifeWorks), we identified potential study participants on the basis of their medication use, diagnoses, and current BMI. A personalized study invitation letter was prepared for each potential participant and sent to their primary care provider, psychiatrist, or psychiatric nurse practitioner, to review for suitability for the study and to co-sign if appropriate. Clinicians returned letters to study staff for mailing to potential participants. The letters provided details about the study, a toll-free study phone number, and a prepaid postcard to indicate interest in participating or to decline further contact. Letters were followed with phone calls by study recruitment staff who provided additional study details, answered questions, and conducted a brief screening. Those who met preliminary qualifications for the study were scheduled for the first screening visit.

At the first screening visit, potential participants attended a group orientation session where they received an overview of the study and had a chance to ask questions. Those who remained interested consented to having their height and weight measured. BMI was then calculated, inclusion and exclusion criteria reviewed, and eligibility established. Eligible participants completed a questionnaire and scheduled a second screening and randomization visit at which a baseline fasting blood specimen was drawn.

At the second screening visit, participants arrived having fasted for at least eight hours, had their blood drawn and their blood pressure and waist circumference measured. Individuals who had not been fasting were rescheduled. All individuals received reminder post-cards prior to the visit that included fasting and medication instructions (i.e., directions to postpone taking diabetes medications until after the fasting blood draw) and asking them to wear loose clothing to aid measurements. Participants also received reminder telephone calls the day before the visit. Following the blood draw, participants were provided with a snack and asked to take any medications at that time if they could not do so prior to the blood draw. They were then randomized to either intervention or usual care groups using a blocked, stratified procedure to ensure that group assignments remained balanced across gender and baseline BMI (27–34.9 and ≥ 35) within each site. Clinic staff involved in collecting data were blinded to participant assignment. Once group assignment was determined, non-clinic staff informed the participant and provided appropriate information about upcoming study activities. Intervention participants were notified when the group intervention was expected to begin; usual care participants were scheduled for the first follow up assessment, e.g. after 6 months. Usual care participants had no other obligation to the study and were free to initiate any weight loss effort on their own. We assessed all self-reported weight loss efforts at each follow-up for both the intervention and usual care participants.

### Tailored lifestyle intervention

The STRIDE intervention was based on the NHLBI-funded PREMIER lifestyle intervention, DASH diet arm [30]. It was designed to promote weight loss and reduce obesity-related risks, including diabetes risk, through dietary changes, moderate calorie reduction, and increased energy expenditure. Intervention targets were based on clinical practice guidelines established for obesity treatment for individuals at increased risk for cardiovascular disease [32,33]. The intervention built upon prior research [27], behavior change theories [34,35], and motivational theory [36-39] to enhance self-efficacy and promote long-term behavior change. Adaptations to content and implementation made specifically to suit this population have been described elsewhere [25]. The STRIDE intervention manual is available for download [40].

STRIDE began with a 6-month intensive group counseling phase followed by a less-intensive 6-month maintenance phase that included both group and individual contacts. Throughout both phases, implementation strategies included: frequent contacts, participant-centered group and individual facilitation approaches, and individual contacts that tailored the intervention to the participant’s preferences (as components of group meetings and separately, when needed). The program also targeted participants’ readiness to change, and encouraged group interactions that facilitated problem solving and social support, provided support for appropriate goal setting, and facilitated the acquisition of new information and skills for behavior change. Group leaders presented examples of behavioral options and used decisional balance approaches [41] to assist participants in moving toward action and setting new behavioral goals, paying careful attention to the cultural appropriateness of the program for minority participants. Sessions were designed to actively engage participants in small-group activities that fostered program ownership and allowed individual interaction with other participants and facilitators. Groups were co-led by two facilitators with complementary backgrounds; one with training as a mental health counselor, the other with training in nutritional interventions, though not a registered dietician. Having two interventionists provided flexibility during group sessions, however, and their differing backgrounds provided knowledge for managing a full range of problems and questions. All interventionists attended an initial two-day training meeting on the general intervention approach, and an additional two-day training meeting in the use of motivational interviewing techniques. In Year 1, an additional one-day training (webcast) addressed methods for presenting materials to individuals with mental illness, including those with cognitive and literacy limitations. Follow-up one-day trainings were held for all interventionists every six months. Trainers also provided ongoing supervision and individual training of all interventionists.

#### Intensive intervention (Phase 1)

The core of the intervention program was a series of weekly group meetings designed to achieve a weight loss of 4.5-6.8 kg (10–15 pounds) over a 6 month period. Groups were two hours in length and included 20 minutes of physical activity (walking). Participants received a STRIDE workbook with details on program content, self-assessments and goal-setting procedures, booklets for self-monitoring of food and activity, cooking and meal planning guides with recipes, a copy of the Calorie King book [42] to help them learn about the calorie content of typical foods, and a resistance band for strength training. Ancillary materials can be downloaded at the study website [43].

Using a manualized protocol, facilitators promoted the following specific strategies for achieving changes in behavior, activity level, and weight: 1) self-monitoring of diet and physical activity, 2) developing personalized diet and physical activity plans, 3) moderately reducing calories, 4) reducing portion sizes, substituting alternative foods, and modifying meals so that they are lower in calories and fat, 5) focusing on fruits and vegetables and increasing low-fat dairy products and fiber intake (DASH diet [44]), 6) increasing physical activity, 7) identifying situations that trigger poor diet or physical activity choices, and developing and rehearsing specific plans of action to deal with those situations, and 8) graphing behavioral progress and individual weight change.

Participants were encouraged to routinely monitor food intake, calories, and physical activity; to set reasonable short-term goals; formulate specific plans of action to achieve those goals; and to develop reinforcement and social support for carrying out each major element of the plan. Participants were also asked to keep records of servings of fruits and vegetables, servings of low-fat dairy products, and sodium and fat intake. These records were used by participants and interventionists to assess progress. Because this program placed a strong emphasis on increasing moderate-intensity physical activity, interventionists also helped participants determine how best to fit physical activity into their daily lives, taking into account each participant’s initial motivation, current activity patterns, and weekly progress. The goal was to get each participant to engage in 180 minutes per week, or about 25 minutes per day, of moderate physical activity—primarily walking. Participants were asked to record minutes of physical activity each day, as well as hours slept each night. Sleep hygiene was a module added to the original PREMIER intervention when modifications were made for this population. Other modifications included sessions focusing on stress management, advance planning for anticipated episodes of mental illness, empowering participants to engage in discourse with their prescribers regarding their medication and weight-related concerns, and tips on eating healthily on a restricted income.

Our experience has shown that willingness to make behavior changes shifts frequently during long-term weight loss intervention programs. Recognizing this reality, intervention techniques allowed group facilitators to help participants tailor weekly goals and action plans to their current stage of change. Although general guidelines for intervention components were provided by the interventionists (daily self-monitoring of caloric intake, five or more days per week of physical activity, etc.), participants were also encouraged to adjust their personal goals and action plans each week to their immediate situation, setting their own short-term goals each week in consultation with the group facilitator. Participants with no interest in physical activity were not forced to set an exercise goal, although those who declined to set a physical activity goal for several consecutive weeks were encouraged to schedule an individual brief session with the group facilitator who worked with them using motivational interviewing techniques [38]. The objective of this counseling session was to encourage reflection on long-term goals, increase motivation to engage in more physical activity, and address barriers to increased activity.

#### Maintenance intervention (Phase 2)

Where the focus of Phase 1 was on weight loss and acquisition of new information and behaviors, Phase 2 focused on maintaining weight loss through problem-solving and motivational enhancement. Because previous research has shown that greater frequency of contacts between participants and providers improves maintenance of weight loss [32], long-term obesity treatment was a priority for the high-risk participants in this study. At the same time, it is also important to recognize the difficulty of maintaining the type of high-intensity commitment required by Phase 1. Thus, Phase 2 consisted of one monthly group meeting to enhance group problem solving and social support, and one low-intensity individual session per month (by phone or e-mail) to support participants’ individualized goals without requiring time and travel to a group session. In these individual contacts, participants set the agenda and the facilitator and participants jointly reviewed the participants’ diet and activity efforts for the previous month, paying particular attention to barriers or difficulties.

### Assessment data collection and measurement

Intervention and usual care groups were assessed at baseline, 6, 12, and 24 months. Laboratory, physiological and anthropometric measures were obtained at each assessment and included: 1) height in meters measured to the nearest 0.1 cm (baseline only), 2) weight in kilograms measured to the nearest 0.1 kg (body mass index was calculated as the Quetelet index [kg/m^2^]), 3) waist circumference, measured over participants’ underwear using inelastic tape at the narrowest part of torso and measured in a horizontal plane without compressing the skin, and 4) blood pressure, measured while participants were seated with legs uncrossed and without talking after a 5-minute rest period, and then again after an additional 30 second rest period. Blood was drawn and all lab panels were collected after an overnight (8–12 hour) fast. Tests included: 5) fasting insulin, 6) fasting plasma glucose, 7) fasting triglycerides, and 8) fasting cholesterol (total, HDL, LDL).

We followed the National Cholesterol Education Program (NCEP) guidelines [45] and defined metabolic syndrome as: Individuals with 3 or more of the following risk factors: a) waist circumference greater than 102 centimeters in men or 88 centimeters in women; b) triglycerides plasma level of 150 mg/dL or greater; c) HDL-cholesterol plasma level less than 40 mg/dL for men or less than 50 mg/dL for women (or taking cholesterol-lowering medications); d) blood pressure of 130/85 mm Hg or greater (or taking antihypertensive medications), and/or e) fasting glucose greater than or equal to 110 mg/dL (or taking glucose lowering medications).

To assess insulin resistance, we calculated the Homeostasis Model Assessment Index for Insulin Resistance (HOMA-IR) as follows: fasting glucose [mmol/L] × fasting insulin [μU/mL]/22.5. This index correlates very highly with the gold standard hyperinsulinemic euglycemic clamp [46].

We included several self-reported measures to describe participants and assess health and functional status and medication use. These measures are shown in Table 1.

**Table 1:** STRIDE measures and data collection schedule

	**BL**	**6 month**	**12 month**	**24 month**
**Physiologic measures**				
Height	x			
Weight	x	x	x	x
Body mass index (BMI)	x	x	x	x
Waist circumference	x	x	x	x
Blood pressure	x	x	x	x
**Laboratory measures**				
Fasting insulin	x	x	x	x
Fasting plasma glucose	x	x	x	x
Fasting triglycerides	x	x	x	x
Fasting cholesterol (total, HDL, LDL)	x	x	x	x
**Self-reported measures**				
Age	x			
Gender	x			
Race	x			
Ethnicity	x			
Education level	x	x	x	x
Medicaid status	x	x	x	x
Medicare disability status	x	x	x	x
Marital status	x	x	x	x
Employment status	x	x	x	x
Annual and monthly income	x	x	x	x
Number of individuals supported by income	x	x	x	x
Income source	x	x	x	x
SF-36 general health subscale [47-49]	x	x	x	x
Behavior and Symptom Identification Scale (BASIS-24) [50]	x	x	x	x
Colorado Symptom Index (CSI) [51,52]	x	x	x	x
Self-reported medications^1^	x	x	x	x
Wisconsin Quality of Life Index (W-QLI) [53-55]	x	x	x	x
Patient Activation Measure (PAM) [56]	x	x	x	x
Body Weight, Image and Self-Esteem Evaluation Questionnaire (B-WISE) [57]	x	x	x	x
Social support scales for eating and exercise behavior [58]	x	x	x	x
Exercise confidence questionnaire [59]	x	x	x	x
Eating habits confidence questionnaire [59]	x	x	x	x
Weight outcome expectancies- ideal and goal weight [60,61]	x	x	x	x
7-day physical activity and sleep log [27]	x	x	x	x
7-day fruit and vegetable consumption [62]	x	x	x	x
Self-reported other efforts to lose weight	x	x	x	x

^1^Self-reported medications were also collected at 18 months.

### Data analysis

#### Effectiveness analyses of primary aims

We will use generalized estimating equations (GEE) [63] to test the effectiveness of the intervention. GEE estimates the population averaged model while accounting for the correlation among observations as a result of having multiple measures from the same person over time. The primary coefficient of interest will be the time by group (control vs. intervention) interaction, which will indicate the degree of differential change across time between the control and intervention groups. We will include age and study site and time-invariant covariates. Use of outcomes-related medications will be included as time-varying covariates (e.g., controlling for metformin usage when testing intervention efficacy on diabetes risk). The GEE models will be based on the normal distribution with identity link, and the working covariance matrix will be specified as exchangeable. An advantage of GEE is that it is able to estimate models when data are incomplete, assuming the data are missing at random and the working covariance matrix is correctly specified [63]. This will allow us to include all participants according to their initial group assignment in the analyses, which is consistent with the intent-to-treat principle.

For every outcome, we will conduct a series of sensitivity analyses by using transformations that improve the normality of the outcome, using a different family and link (e.g., negative binomial with log link) where appropriate, and using an unstructured working covariance matrix.

Finally, we will also examine between-group differences for various conceptualizations for the primary outcome of weight, including percentage weight change, the proportion of participants who are at or below his or her baseline weight, and the proportion of participants who lose at least 5% and 10% of their baseline weight. These will be computed at all follow-up timepoints. These are not intent-to-treat results, as these computations require that data are not missing in order to compute the necessary change scores for deriving these different measures of weight. We will test the difference in percentage weight change between the intervention and control groups using a one-way ANCOVA, and test whether the proportion of participants who are at or below their baseline weight at follow-up is different between the arms using multiple logistic regression. We will include age, site, and medications that are known to affect weight as covariates in all analyses.

#### Analyses of secondary aims

We plan on examining the effects of exercise motivation, dietary motivation, social support and outcomes expectancies on weight change. We will also explore whether ethnicity, gender, mental health diagnostic group, medication type, and metabolic syndrome moderates the treatment effect on weight change. Furthermore, we will examine whether there was an effect of the intervention on patient reported outcomes including body image (Body Weight, Image and Self-Esteem Evaluation Questionnaire - B-WISE), psychiatric symptoms (Colorado Symptom Index - CSI), quality of life (Wisconsin Quality of Life Index - W-QLI), health-related self-efficacy (Patient Activation Measure - PAM), health (SF-36 general health subscale), and functional status (Behavior and Symptom Identification Scale - BASIS-24). Finally, we will evaluate whether dose, defined as the number of intervention sessions and number of telephone and email contacts, is related to change in the outcomes across time among participants who received the intervention.

#### Power analysis of primary aims

We used data from PREMIER DASH diet arm results [30] to estimate power for repeated measures analysis of variance for change in weight. We used repeated measures ANOVA given the lack of well-established methods for estimating power using GEE. Data were available for change at 6 and 18 months. Because reported means and standard deviations were based on completers alone, we adjusted for attrition in our power analyses (although primary analyses will be intent-to-treat). PREMIER reported a 5.9% decrease in weight at 6 months for the DASH diet group and a 1.6% decrease for control (advice only). With an initial total sample size of 280, a 10% (worst case) attrition rate at six months, and alpha level of .05, we have power of .96 to detect a significant group-by-time interaction at 6 months. Using the 18-month values (−1.6% for advice only, -4.4% for DASH) as a conservative estimate for weight change at 12 months, we have power of .87 for the group-by-time interaction term for weight (assuming a 15% worst-case attrition rate). To estimate power at 24 months, we assumed the same rate of weight gain in the DASH group and weight loss from 6 to 18 months in the UC group, consistent with PREMIER. For a 3.6% decrease from baseline in the DASH group and 1.8% decrease for UC at 24 months, we have power of .80 to detect a significant group-by-time interaction at 24 months, assuming 20% attrition. Power for waist circumference, fasting insulin, and insulin sensitivity were based on data reported by Ard [31]. Power for total cholesterol, LDL, and HDL were based on data reported by Obarzanek [64]. Only baseline and 6-month means were available for these variables, so we have computed power to detect a significant group-by-time interaction at 6 months only. For all variables, power exceeds .95 to detect the group-by-time interaction.

#### Implementation and process evaluations

The implementation evaluation included observations of organizational and other facilitators and barriers to implementation; results of this evaluation are described elsewhere [25]. Fidelity assessments documented adherence to the intervention protocol and thus will allow examination of implementation differences across groups. Assessment of intervention fidelity includes both qualitative and quantitative measures.

The study design also included a process evaluation component, which sought to understand participants’ feelings toward, and responses to, the different elements of the intervention and to lifestyle change more generally. Semi-structured interviews lasting approximately 30 minutes were conducted with participants at 3- (n = 38), 9- (n = 35), and 18-months (n = 29) following randomization. Interviewers asked about barriers and facilitators of dietary and exercise changes. Of these, 76% (n = 78) were conducted with intervention participants and 24% (n = 24) with control participants. Interviews were transcribed verbatim and coded using Atlas.ti. Analyses will identify themes related to lifestyle improvements across participants; check coding will assess inter-coder reliability.

#### Cost-utility analysis

The study was designed to conduct a cost-utility analysis and includes estimates of the direct costs, per participant, of implementing the intervention. Relevant costs for these analyses will include the resources necessary to deliver the intervention and the change in care costs incurred by the organization that derive from the intervention. We have included the EQ-5D [65], a brief, self-administered, health-related quality of life measure that assesses mobility, self-care, usual activities, pain/discomfort, and anxiety/depression dimensions. Response patterns yield 243 unique health states, and are commonly used to generate a composite score or index reflecting the preference value (utility) associated with a given health state. We will calculate incremental cost-effectiveness ratios for each intermediate trial outcome, and will further report such a ratio for the improvement in mean EQ-5D scores between the intervention and control groups.

### Baseline characteristics

A total of 200 individuals with serious mental illnesses were randomized to one of the treatment arms. Two participants became ineligible after randomization, one due to pregnancy and one due to an unspecified but serious health problem that caused repeated vomiting and resulted in a significant weight loss. At baseline (Table 2), participant diagnoses were affective psychosis (38%), bipolar disorder (32%), schizophrenia spectrum disorders (29%) and PTSD (2%). Participants were taking an average of 3.2 psychiatric medications and 91% were taking an atypical antipsychotic medication. Study participants were an average age of 47.1 (SD = 10.7) years old. Thirty percent were currently working and 35% reported being disabled. About 31% of the sample had a high school diploma, GED or less, and 43% were married or living with a partner.

**Table 2:** Baseline characteristics of the study participants (N = 200)

**Characteristic**	**n (%)**^**1**^
Inclusion diagnosis	
Affective psychosis	75 (38)
Bipolar disorder	63 (32)
Schizophrenia spectrum disorder	58 (29)
Post-traumatic stress disorder	4 (2)
Gender	
Male	56 (28)
Female	144 (72)
Race^2^	
White	171 (88)
Non-white	24 (12)
Hispanic ethnicity	4 (2)
Education	
Less than high school	15 (8)
High school graduate/GED	46 (23)
Some college/technical school	87 (44)
College graduate	37 (19)
Post graduate	15 (8)
Marital status	
Never married	57 (29)
Widowed/Divorced/Separated	57 (29)
Married	69 (34)
Living with partner	17 (9)
Annual household income	
$0 - $9,999	54 (28)
$10,000 - $29,999	58 (30)
$30,000 – $49,999	34 (17)
$50,000 or higher	49 (25)
Number of people supported by income, mean (SD)	2.0 (1.3)
Employment status	
Currently working	59 (30)
Disabled	71 (36)
Retired, unemployed, student, homemaker, temporarily laid off, or other	70 (35)
Atypical antipsychotic medication use	182 (91)
Medications taken that affect weight	
≥1 that cause slight/moderate weight loss	77 (39)
≥1 that do not affect weight	168 (84)
≥1 that cause slight/moderate weight gain	21 (11)
≥1 that cause severe weight gain	128 (64)
Mood stabilizer medication use	97 (49)
Anti-depressant medication use	33 (17)
Smoked in the past year	65 (33)
Self-reported diabetes diagnosis	
No	145 (73)
Yes	48 (24)
Don’t know	7 (4)
Self-reported hypertension diagnosis	
No	114 (57)
Yes	70 (35)
Don’t know	15 (8)
	**Mean (SD)**
Age—years	47.1 (10.7)
Weight—kg.^3^	107.7 (25.1)
Waist circumference—cm.^3^	113.9 (18.7)
Body mass index^4^	38.3 (8.3)
Blood pressure: systolic—mmHg	119.2 (14.7)
Blood pressure: diastolic—mmHg	79.4 (10.1)
Lipids	
Triglycerides—mg/dL	188.0 (138.6)
LDL—mg/dL	101.4 (32.9)
HDL—mg/dL	45.8 (12.7)
Total cholesterol—mg/dL	181.6 (39.7)
Fasting glucose—mg/dL	108.9 (32.5)
Fasting insulinu—U/mL	13.0 (11.9)
Goal weight differential—kg.^5^	−31.7 (18.5)
Very likely to achieve goal weight, n (%)	33 (17)
Ideal weight differential—kg.^6^	−38.2 (21.5)
Very likely to achieve ideal weight, n (%)	21 (11)
Number of psychiatric medications	3.2 (1.5)
Psychiatric measures	
CSI score^7^	19.3 (11.4)
BASIS-24 score^8^	1.37 (0.68)
SF36-GH score^9^	53.2 (21.3)
PAM13 (normed) score^10^	62.4 (16.0)
B-WISE score^11^	21.9 (3.6)

^1^Percentages reported use number of valid responses in the denominator. Percentages may not sum to 100% because of rounding.

^2^Race and ethnic groups were self-reported.

^3^To convert values for weight to pounds, multiply kilograms by 2.2. To convert values for centimeters to inches, multiply centimeters by 0.39.

^4^Body Mass Index is the weight in kilograms divided by the square of height in meters.

^5^The difference between self-reported *goal* weight and observed weight at baseline.

^6^The difference between self-reported *ideal* weight and observed weight at baseline.

^7^Colorado Symptom Index.

^8^Behavior and Symptom Identification Scale 24-item version.

^9^SF-36 General Health Subscale.

^10^Patient Activation Measure.

^11^Body Weight, Image and Self-Esteem Evaluation Questionnaire (B-WISE).

The sample had an average baseline weight of 107.7 kilograms (SD = 25.1), waist circumference of 113.9 centimeters (SD = 18.7), BMI of 38.3 (SD = 8.3) and 24% of the sample reported a diabetes diagnosis. Differences between observed weight and goal or ideal weights were 31.7 kilograms (SD = 18.5) and 38.2 kilograms (SD = 21.5) respectively. Baseline physiological measures included mean blood pressure (SBP/DBP) measurements of 119.2 (SD = 14.7)/79.4 (SD = 10.1); 35% reported a hypertension diagnosis with 11% of people taking antihypertensive medications. Average lipid levels (mg/dL) were: a) triglycerides 188.0 (SD = 138.6), ranged from 43 to 1145; b) LDL 101.4 (SD = 32.9) and ranged from 17 to 185; c) HDL 45.8 (SD = 12.7) and ranged from 22 to 89; and d) total cholesterol 181.6 (SD = 39.7) and ranged from 50 to 324. Average fasting glucose levels were 108.9 (SD = 32.5) and ranged from 24 to 289. Average fasting insulin levels were 13.0 (SD=11.9) and ranged from 2 to 99. Psychiatric measures at baseline include a mean CSI score of 19.3 (SD = 11.4, possible scores range from 14–70 with higher scores indicating more frequent symptoms), and a BASIS-24 mean score of 1.37 (SD = 0.68, possible scores range from 0 to 4, higher scores represent higher levels of difficulty in symptoms and functioning). Patient activation, measured by the PAM score averaged 62.4 (SD = 16.0; scores between 55.2-67.0 are associated with beginning to take action in managing one’s health). Body image, measured with the B-WISE, averaged 21.9 (SD = 3.6, potential scores range from 12–36 with higher scores indicating a more positive body image).

## Discussion

Though lifestyle interventions aimed at reducing weight have been adapted for individuals with serious mental illnesses, their effectiveness has been limited. Even effective interventions have only resulted in modest weight loss for a minority of participants. There is a critical need for more evidenced-based programs shown to benefit a greater proportion of individuals, produce clinically meaningful weight loss, reduce obesity-related cardiovascular risks, and improve physical fitness. Moreover, it is important that such interventions can be implemented in community-based settings.

STRIDE is a lifestyle intervention modified for the unique needs of overweight individuals taking antipsychotic medications and delivered in community mental health and integrated care settings. Despite the challenges of implementing a rigorous weight reduction intervention in any population, especially a population with SMI, we are encouraged by our ability to recruit and retain participants in STRIDE. To succeed in retaining individuals in the study we employed several strategies for accommodating the life circumstances of this disadvantaged population. We met in several locations to minimize transportation, we provided reminders prior to group meetings and study visits. We also called individuals who missed group meetings and offered make-up sessions.

This study was designed to test the effectiveness of this intervention to reduce weight, fasting insulin levels, total cholesterol and LDL cholesterol and increase insulin. The study was also designed to explore potential mediators and moderators of the intervention’s effects, to examine barriers and facilitators of behavior change, to assess issues with implementing the intervention in this population and to provide cost-effectiveness data in order to inform future research and provide decision makers with information needed for future program adoption decisions. The results of this study will contribute to a better understanding of how to assist individuals with mental illnesses to manage their weight and improve their overall health.

## Abbreviations

BMI: Body-mass index; GEE: Generalized estimating equation; HOMA: Homeostasis Model Assessment; KPNW: Kaiser Permanente Northwest; PTSD: Post-traumatic stress disorder; GED: General equivalency degree; WQLI: Wisconsin Quality of Life Index; CSI: Colorado symptoms index; BASIS: Behavior and Symptom Identification Scale; SMI: Serious mental illness; PAM: Patient Activation Measure; B-WISE: Body Weight, Image and Self-Esteem Evaluation; LDL: Low-density lipoprotein; HDL: High-density lipoprotein.

## Competing interests

The authors declare that they have no competing interests.

## Authors’ contributions

CG conceived of the design of the study and participated in the design of the intervention, the development of the statistical analysis plan, and had overall responsibility for the study. NP helped with the study design and analysis plan. BJHY participated in the coordination of the trial and the implementation of the intervention. ML helped with analysis plan and was responsible for data presented. All authors helped to draft the manuscript, read and approved the final version.

## Pre-publication history

The pre-publication history for this paper can be accessed here:

http://www.biomedcentral.com/1471-244X/13/238/prepub
